# Understanding organisational and nursing behaviour changes associated with a therapeutic engagement improvement tool in acute mental health inpatient settings: A qualitative analysis

**DOI:** 10.1016/j.ijnsa.2024.100180

**Published:** 2024-01-23

**Authors:** Francesca Taylor, Sarah Galloway, Kris Irons, Lorna Mess, Laura Pemberton, Karen Worton, Mary Chambers

**Affiliations:** a School of Nursing, Allied and Public Health, Kingston University, Kingston Upon Thames, Thames KT2 7LB, United Kingdom; b South West London and St George's Mental Health National Health Service Trust, London SW17 7DJ, United Kingdom; c Priory Group, Cheadle SK8 3DG, United Kingdom; d North East London National Health Service Foundation Trust, Rainham, Essex RM13 8GQ, United Kingdom; e Southern Health National Health Service Foundation Trust, Southampton SO40 2RZ, United Kingdom; f Cumbria, Northumberland, Tyne and Wear National Health Service Foundation Trust, Newcastle upon Tyne NE3 3XT, United Kingdom

**Keywords:** Behaviour, Mental health nursing, Nursing staff, Patient engagement, Quality improvement, Therapeutic

## Abstract

**Background:**

Enhancing the quality of therapeutic engagement between nurse and service user is related to positive impact on care, safety, and recovery outcomes. Achieving improved therapeutic engagement remains challenging in the acute mental health inpatient setting, characterised by complex social processes and contextual features that constrain behaviour change. The Therapeutic Engagement Questionnaire is an evidence-based tool co-produced with service users and nurses to improve therapeutic engagement.

**Objectives:**

The objectives of this quality improvement project were to identify the organisational and nursing behaviour changes associated with the Therapeutic Engagement Questionnaire and to understand the active behaviour change ingredients of the improvement tool and how they exert their influence.

**Design:**

A qualitative multi-site case study design in which data were collected from study site field notes and document review.

**Setting:**

Four acute mental health inpatient case study sites in England.

**Methods:**

Data referencing Therapeutic Engagement Questionnaire-linked behaviour change in project meeting field notes and documents from each study site were analysed using an inductive and deductive approach with thematic analysis. The Capability Opportunity Motivation-Behaviour model was employed as a theoretical framework.

**Findings:**

The therapeutic engagement tool had the capacity to prompt behaviour change across all three components of the behaviour change model: Capability – through nurses sharing good therapeutic engagement practice and use of statements in the questionnaire to build nurses’ knowledge and skills; Opportunity – through organisational barriers being addressed and ward-level practice and culture changes; Motivation – through nurses’ awareness of their influence on service user recovery, nurses’ alertness to their therapeutic work, and connections between the therapeutic engagement tool and nursing core values. However, the tool did not accord with the values of some nurses, reported to be unmotivated by the recognition it gave their profession for contribution to service user recovery. In sites evidencing more prominent behaviour change, senior leader and ward-level agents of change played a valuable facilitative role.

**Conclusion:**

The therapeutic engagement tool had the potential to prompt behaviour changes at organisation and ward level and to the ways individual nurses therapeutically engage with service users, helping strengthen therapeutic engagement practice. Leadership at senior organisational and ward level was important to address contextual barriers to change. The project resulted in a conceptual framework to explain and understand the behaviour change techniques and functions linked to the therapeutic engagement tool. Longevity of the behaviour changes and their impact on service user quality of care requires future evaluation.

**Tweetable abstract:**

A therapeutic engagement tool can prompt organisational and nursing behaviour change in acute mental health inpatient settings.

**What is already known about this topic**

• Poor quality therapeutic engagement between nurse and service user is associated with negative care and safety and recovery outcomes, yet therapeutic interactions remain sub-optimal in the acute mental health inpatient setting.
• The distinct social processes and contextual features characterising acute mental health inpatient wards provide constraining barriers to changing nursing behaviour associated with therapeutic engagement.

Alt-text: Unlabelled box

**What this paper adds**

• A therapeutic engagement tool, the Therapeutic Engagement Questionnaire, can stimulate positive behaviour changes around therapeutic engagement in acute mental health inpatient settings, especially where there are strong agents of change at senior and ward level.
• Theoretical framing of the behaviour change techniques associated with the therapeutic engagement tool provides insights and understanding of how the tool can address the different components of therapeutic engagement behaviour: capability (psychological), opportunity (physical and social), and motivation (reflective and automatic).

Alt-text: Unlabelled box

## 1 Introduction

Reducing the unmet care needs of acute mental health service users is a growing concern internationally (Patel et al., 2018; Johnson et al., 2022). Significant divergence exists between the self-reported care needs of service users and the help and support they receive in practice (Cutliffe et al., 2015). Extant evidence presents an inhospitable picture of the acute inpatient ward environment, with service users reporting their ward experiences as unsafe, disturbing, and discriminatory (Johnson et al., 2022), while lacking warmth, respect, and person-centred care (Staniszewska et al., 2019; McAllister et al., 2021a). Accordingly, United Kingdom (UK) and international policy increasingly focuses on the provision of improved safe and accessible inpatient care; the importance of therapeutic communication and engagement between nurse and service user in this context is often highlighted (Department of Health and Social Care, 2023; National Mental Health Commission, 2020; Organisation for Economic Co-operation and Development (OECD), 2021). There is robust evidence that improving the quality of therapeutic engagement between nurse and service user is associated with beneficial impact on care, safety, and recovery outcomes (Hartley et al., 2020). Conversely, a poor therapeutic relationship is associated with negative clinical outcomes (Bolsinger et al., 2020). Furthermore, the therapeutic alliance has been identified as related to successful outcome independent of service user characteristics and treatment processes (Flückiger et al., 2020).

There is no universally agreed upon definition of therapeutic engagement in acute inpatient settings (McAllister et al., 2019), although concepts, including ‘healing’, ‘benefit', ‘empowerment’, and ‘restorative’, are consistently highlighted in associated discourse (Chambers et al., 2021). Therapeutic engagement has been described as a partnership relationship between the registered mental health nurse and service user with shared decision-making, recovery-focused goals based on mutual trust, respect, and negotiation, enabling service users to problem solve and enhance their coping capacity (Chambers, 1992; Chambers et al., 2021). Intrinsically, therapeutic engagement is a process incorporating the construction and maintenance of a strong interpersonal therapeutic alliance through task and goal agreement. Some authors have drawn a distinction between a therapeutic alliance and a strong relational bond, in recognition of the therapeutic benefit of relational connectedness (Cutliffe et al., 2015). However, the term therapeutic alliance has tended to become interchangeable with the term therapeutic relationship (McAndrew et al., 2014).

Given the multifaceted nature of therapeutic engagement, it has been argued that consideration should be given to the influence of both the therapeutic environment and atmosphere and one-to-one therapeutic sessions between the mental health nurse and service user (Chambers et al., 2021). In relation to one-to-one interpersonal interactions, McAllister et al. (2019) identified five essential components of therapeutic engagement: understanding the person and their experiences, facilitating growth, therapeutic use of self, choosing the right approach, and authoritative versus emotional containment.

Factors identified in the literature as contributing to a positive therapeutic engagement environment from the service user perspective include nurse willingness to listen and understand the individual and their illness; positive attitudes; care offered with compassion, dignity, and respect; and shared decision-making (McAllister et al., 2019; McAndrew et al., 2014). From the mental health nurse perspective, higher-quality therapeutic relationships are associated with nurse practice environments that include resources, information, and staff support (Roviralta-Vilella et al., 2019). Despite this body of evidence, therapeutic engagement on acute inpatient wards remains sub-optimal. Service users consistently criticise the absence of good therapeutic interpersonal relationships and seek improved therapeutic engagement (Cutliffe et al., 2015).

Although evidence-based health care is widely accepted as a quality standard of mental health practice (Le Boutillier et al., 2015) and evidence-based practice is known by inpatient mental health nurses to improve the development of therapeutic relationships (Moreno-Poyato et al., 2021), institutional forces appear more powerful in determining whether and what evidence is used (Martin and Williams, 2019). Alongside the complex needs of service users, the acute inpatient environment is characterised by high workloads, poor staff-to-service user ratios, and ineffective crisis handling, with administrative systems and tasks prioritised organisationally over therapeutic support (Wykes et al., 2018). Few interventions have been introduced on acute inpatient wards to improve the therapeutic environment (Johnson et al., 2022) or to assist nurses in therapeutic engagement (McAllister et al., 2021a; McAllister et al., 2021b). Some interventions have been implemented to protect engagement time, enabling contact time and uninterrupted interactions between nurses and service users (Dodd et al., 2018; Molin et al., 2018). There is some evidence from England and internationally that these interventions increase opportunities for therapeutic engagement (Dodd et al., 2018; Molin et al., 2018); however, no intervention has been reported to improve the quality of therapeutic engagement or service users’ satisfaction with their care (McAllister et al., 2021a). Any impacts on the quality of care appear to be contingent on the capacity, opportunity, and motivation of nurses to engage with service users (McAllister et al., 2021a).

To maximise effectiveness, an intervention needs to be adopted by its target and result in behaviour change (Davis et al., 2015). This is especially challenging in acute inpatient wards, distinguished by distinct social and cultural processes and contextual features that provide constraining barriers to change, including risk-adverse cultures with high potential for disruption through violence and aggression; limited resources, particularly staff and beds; and fast discharge (Laker et al., 2014; Raphael et al., 2021). Additionally, there are increasing numbers of service users with higher levels of acuity and requiring compulsory detention (Wyder et al., 2015; Wykes et al., 2018).

Healthcare interventions in general have been more successful in stimulating change if they address a range of mechanisms, given the complex behavioural interactions between people, organisational structures, and processes (Michie et al., 2011a; Marshall et al., 2017). This is similarly likely to apply to interventions for improving therapeutic engagement in acute inpatient wards, given the lack of clarity around the different complex variables that can contribute to therapeutic engagement in this setting (Chambers et al., 2017) and an absence of shared descriptive language, compounded by nurses often struggling to articulate what they do in relation to therapeutic engagement with service users (McAllister et al., 2021a). Behaviour changes are also more likely if service users are involved in the intervention development (Brett et al., 2014).

The Therapeutic Engagement Questionnaire was co-produced with registered mental health nurses (registered as fit to practice as mental health nurses) and service users as a multidimensional therapeutic engagement improvement tool for incorporation into routine clinical practice in acute inpatient settings. It was designed with the objectives of quantifying and recognising how registered mental health nurses therapeutically engage with service users in delivering evidence-based therapeutic engagement into clinical practice to achieve outcomes of enhanced service user care and recovery (Chambers et al., 2021). Earlier published articles provide details of the Therapeutic Engagement Questionnaire's development (Chambers et al., 2017; Chambers et al., 2019) and implementation into practice (Taylor et al., 2022). There are service user and registered mental health nurse versions of the questionnaire (Kingston University London, 2023a). In each version, therapeutic engagement is scored across two different contexts: one-to-one registered mental health nurse and service user interactions and the overall ward environment and atmosphere. Twenty therapeutic engagement statements are included with pre-set answer options using a 4-point Likert response scale. There is a specific scoring system; the higher the score, the better the therapeutic engagement.

This paper focuses on identifying and understanding the organisational and nursing behaviour changes associated with the Therapeutic Engagement Questionnaire, in the quality improvement tool's early ‘innovation’ phase (Marshall et al., 2017), prior to any measurement of its effect on service user care and recovery outcomes. Quality improvement tools may fail without an understanding of the mechanisms of change needed to support improvement and achieve the intended outcomes (Marshall et al., 2017). Such understanding can support sustainable change (Michie, 2011a), helping guide the expectations, preparations, and adaptations of implementing organisations. To our knowledge, there is no other quality improvement project that seeks to understand the features of a therapeutic engagement tool that can influence behaviour change in practice in multi-site acute mental health inpatient settings. The objectives of this project were to identify the organisational and nursing behaviour changes associated with the Therapeutic Engagement Questionnaire in acute mental health inpatient settings and to understand the active behaviour change ingredients of the improvement tool and how they exert their influence.

## 2 Methods

### 2.1 Project design

A qualitative multi-site case study design was used, in which data were collected from case study site field notes and document review (Holloway and Galvin, 2016). This data combination enabled a pragmatist approach with focus on understanding real-world issues and production of actionable knowledge of practical relevance (Kelly and Cordeiro, 2020).

A multi-site case study approach was employed to analyse the data within and across case study sites and enable understanding of the similarities and differences between sites (Yin, 2018). This method also allowed for knowledge to be developed about site-specific and shared behavioural techniques that may be involved in change and by which the therapeutic engagement tool achieves its influence. Guidance was used for ensuring quality and transparency in methodological approach (O'Brien et al., 2014).

A convenience sample of four case study sites provided data. The case study sites were selected from a total of seven sites that voluntarily implemented the Therapeutic Engagement Questionnaire in response to a letter sent by the project lead through the National Mental Health Nurse Directors Forum. Selection for in-depth case studies was on the basis of their provision of site-specific documents referencing any commentary, observations, or reports on use of the Therapeutic Engagement Questionnaire during the fieldwork period June 2020 to October 2021. Only staff employed by the site organisations distributed the Therapeutic Engagement Questionnaire and collated and analysed the data. The selected sites offered regional spread across England and variation in organisation, rural-urban location, and area population ethnicity (Table 1). Three sites were National Health Service Mental Health Trusts and one a private provider.

**Table 1:** Case study site characteristics.

Case study site	Rural/urban location^a^	Number of participating wards	Area population ethnicity^b^	Region of England
1	Urban with major conurbation	3	Asian 47%, White 35%, Black 8%	Southeast
2	Urban with major conurbation	3	White 68%, Asian 12%, Black 10%	Greater London
3 Location A	Urban with city and town	1	White 52%, Black 18%, Asian 12%	Southeast
Location B	Urban with major conurbation	1	White 81%, Asian 11%, Black 3%	Greater London
4	Urban with significant rural	4	White 93%, Asian 4%, Black 1%	Northeast

a Office for National Statistics, 2015.

b Ethnicity Facts and Figs Service, 2022.

Two university researchers (FT, MC), a research associate, and a professor provided project support to case study sites using the Therapeutic Engagement Questionnaire during the fieldwork period. The support mechanism was project facilitation meetings involving the researchers and key stakeholders, including nurse directors, senior clinicians (nurse consultants, quality improvement and innovation managers, matrons, band 8 nurses (a high-level UK National Health Service grade and salary level), ward managers, and nurses. The format and frequency of the meetings was described in detail in an earlier paper (Taylor et al., 2022). All meetings were held online because the fieldwork took place during the COVID-19 pandemic. This meant that the university project researchers could not directly observe the ward cultures and contexts.

The Capability Opportunity Motivation-Behaviour model, which forms the core of the Behaviour Change Wheel (Michie et al., 2011a), was used as a framework for data analysis and interpretation. According to this evidence-based model, any behaviour change is the result of an interaction between three components — capability, opportunity and motivation — each component being able to influence behaviour both independently and collectively (Michie et al., 2013). Capability encompasses the psychological (required knowledge and skills) and physical (required capacity to engage in the behaviour). Opportunity can be physical (environmental resources) and social (societal influences such as cultural norms and interpersonal factors). Motivation relates to the mental activity that provokes behaviour, which can be automatic (emotions, wants, needs, habits) and reflective (beliefs, plans, intentions) (Michie et al., 2011a). These components are linked to a range of functions that categorise the different ways of instigating behaviour change: education, coercion, training, restriction, environmental restructuring, modelling, enablement, incentivisation, and persuasion. Complementing the framework is the Behaviour Change Techniques Taxonomy, which describes specific behaviour change techniques that make up the content of an intervention, the active components that effect change. Used in evaluations, the model enables insight and understanding into the components of an intervention that are effective in changing behaviour (Michie et al., 2011b).

The Therapeutic Engagement Questionnaire was implemented as a previously validated service improvement tool (Chambers et al., 2019). This project was part of local case study site quality improvement projects and did not require institutional ethical approval. The therapeutic engagement improvement tool was signed off by the nursing directors of the implementing organisations, and the project was carried out in accordance with the principles of the Declaration of Helsinki.

Considerable attention was given by each organisation implementing the therapeutic engagement improvement tool to ensure safety for service users and registered mental health nurses. This included providing assurance of anonymity and ward managers, followed by senior nursing staff checking the completed anonymous questionnaires for any safeguarding issues and responding to any concerns raised.

### 2.2 Data collection

Data were collected by the project researchers from field notes collected during the project facilitation meetings and from local case study site documents. Two project researchers (FT, MC) attended each facilitation discussion meeting. One of these project researchers simultaneously participated in the meetings while jotting down notes on dialogue, atmosphere, behaviour, and decisions. These jottings were expanded later into field notes. Requests were made to stakeholders of the case study sites for available documents that referenced the Therapeutic Engagement Questionnaire. These site-specific documents (*n*=18) included project facilitation discussion meeting notes and presentation slides from internal staff presentations and national conferences. All data were anonymised and safe storage ensured in line with Kingston University London best practice principles of data protection and archiving (Kingston University London, 2023b).

### 2.3 Data analysis

Data were analysed in three stages involving inductive (data driven) and deductive (theory framed) analysis (Pope and Mays, 2020). In stage one, field notes and documents from each of the case study sites were analysed for any reference or commentary on behaviour change associated with the Therapeutic Engagement Questionnaire and these data extracted to a spreadsheet. Data were then coded and analysed thematically (Boyatzis, 1998), using an inductive approach informed by the project objectives. One researcher (FT) familiarised themselves with the data and developed an index coding, subsequently iterated and refined through discussion with a second researcher (MC), until all data were coded and organised into categories. Where data did not fit generated themes, new categories were developed or existing ones refined. Stage two consisted of a thematic cross-case analysis undertaken by one researcher (FT), supplemented by discussion with a second researcher (MC) to identify and explore similar cross-cutting themes and where there were divergent case-specific exceptions. A further stage of analysis was undertaken by two researchers that involved deductive exploration of the identified inductive cross-cutting themes in the context of the Capability Opportunity Motivation-Behaviour model (Michie et al., 2011a).

## 3 Findings

The themes and sub-themes resulting from the cross-case data analysis, organised around the Capability Opportunity Motivation-Behaviour model domains of capability, opportunity, and motivation, are discussed below. Table 2 shows the behaviour change techniques and functions of the Therapeutic Engagement Questionnaire identified within the themes and sub-themes and linked to the Capability Opportunity Motivation-Behaviour model.

**Table 2:** The Therapeutic Engagement Questionnaire behaviour change techniques and functions linked to the Capability Opportunity Motivation-Behaviour model.

Capability Opportunity Motivation-Behaviour construct Michie et al. (2011a)		Behaviour change techniques Michie et al. (2013)	Study sub-themes	Functions
Capability	Psychological	Review behaviour goals	Use of the Therapeutic Engagement Questionnaire to build knowledge and skills	Education: Local study site Therapeutic Engagement Questionnaire data showed variation in service users’ scores for registered mental health nurses’ therapeutic engagement work, prompting recognition some nurses deficient in knowledge and skills, and interest in training.
		Instruction how to perform behaviour		
		Goal setting		
				Training: Therapeutic engagement statements in the Therapeutic Engagement Questionnaire used by ward managers to train nursing staff in building therapeutic relationships and to set goals for good therapeutic engagement practice.
		Modelling of the behaviour	Sharing of good practice	Modelling: Registered mental health nurses working on wards that achieved better service user scores in relation to therapeutic engagement shared their therapeutic engagement practice with nurses working on poorer scoring wards.
		Demonstration of the behaviour		
				Enablement: Good practice identified by local study site Therapeutic Engagement Questionnaire data and shared with registered mental health nurses provided effective methods to develop and sustain therapeutic engagement.
Opportunity	Physical	Restructuring the physical environment	Organisational barriers confronted	Environmental restructuring: The Therapeutic Engagement Questionnaire acted as a consciousness raiser as to organisational barriers faced by registered mental health nurses in delivering the therapeutic support wanted by service users, prompting organisational change to establish therapeutic engagement as a priority task.
		Action planning		
				Enablement: Registered mental health nurses facilitated to spend more time with service users by giving less focus to completing electronic service user records and less time to multi-disciplinary team meetings and ward reviews.
	Social	Restructuring the social environment	Ward-level practice and culture changes	Environmental restructuring: In response to a local study site's Therapeutic Engagement Questionnaire data showing service users did not know their ‘named nurse’, new practice models introduced increasing service user awareness of the therapeutic engagement role of their ‘named nurse’. Questionnaire data showing low levels of service user engagement in care planning prompted introduction of nursing practice improvements to encourage service user engagement.
		Social support		
Opportunity	Social	Prompts/cues	Ward-level practice and culture changes	Enablement: Discussion among ward managers and registered mental health nurses about Therapeutic Engagement Questionnaire data encouraged registered mental health nurses to give attention to their therapeutic engagement practice.
Motivation	Reflective	Information about others’ approval	Awareness of registered mental health nurses’ influence on service user recovery	Education: Local study site Therapeutic Engagement Questionnaire data highlighted the value of registered mental health nurses’ therapeutic engagement behaviour to service user recovery.
		Information about health consequences		
				Persuasion: Credibility of the Therapeutic Engagement Questionnaire as a data source persuaded registered mental health nurses, nurse directors, and senior clinicians of the accuracy of data showing the influence of registered mental health nurses on service user recovery.
		Credible data source		
		Feedback on behaviour		
				Persuasion: Feedback on differences between service users and registered mental health nurses in how they scored the registered mental health nurse contribution to service user recovery, prompted reflective ward-level discussions on nurses’ role in supporting service user recovery.
		Comparison of behaviour	Alertness to therapeutic work	Persuasion: Local study site Therapeutic Engagement Questionnaire data highlighted variation between service users in how they rated the contribution of their named nurse to their recovery, prompting ward-level reflective discussion among nurses on how these differences might be addressed.
		Discrepancy between current behaviour and goal standard		
	Automatic	Identity associated with changed behaviour	Connection with nursing values	Reattribution: Alignment of the Therapeutic Engagement Questionnaire with the core registered mental health nursing values of care and recovery encouraged registered mental health nurses to reattribute these values to their work.
				Persuasion: The Therapeutic Engagement Questionnaire reinforced the importance of the core registered mental health nursing values of care and recovery.

### 3.1 Capability

#### 3.1.1 Use of the therapeutic engagement questionnaire to build knowledge and skills

A frequently-raised challenge in project facilitation meetings in all case study sites was the variation in how service users scored the therapeutic engagement work of registered mental health nurses, highlighted in the Therapeutic Engagement Questionnaire data collected by each site. Discussion on these findings among senior stakeholders (nurse directors and senior clinicians), ward mangers, and nursing staff prompted recognition that some registered mental health nurses were deficient in the training and skills needed to deliver good therapeutic engagement. For example, stakeholders in one site talked about nurses having insufficient knowledge and understanding about therapeutic interactions they might employ to develop a therapeutic relationship. In another site, stakeholders reported an *‘increase in requests by nurses to do a more therapeutic engagement based CPD* (continuing professional development) *course*’. (Project facilitation meeting – nurse director/senior clinician, ID 01).

In response, individual ward managers from different sites were stimulated to use the therapeutic engagement statements in the Therapeutic Engagement Questionnaire to foster knowledge of therapeutic interactions that could be employed to build therapeutic relationships. For example, one ward manager used the questionnaire statements in inductions for new nurses, in parallel introducing service users to the Therapeutic Engagement Questionnaire on their admission, explaining that this is what they should expect as a service user. Use of the Therapeutic Engagement Questionnaire as a template for good practice was felt to have *‘enabled better and shared expectations between staff and service users on admission’* (Document – nurse director/senior clinician, ID 16). Another ward manager referenced the questionnaire statements in clinical service supervisions.

*The data will inform training and professional development for the nursing team, (and) is of value within clinical service supervision.* (Document – nurse director/senior clinician, ID 04).

#### 3.1.2 Sharing of good practice

Teaching was supplemented in some case study sites by the sharing of good practice. Stakeholders in these sites reported Therapeutic Engagement Questionnaire data in project facilitation meetings and site documents that showed variation between wards in how service users scored the quality of therapeutic engagement and the extent to which it contributed to their recovery. Identification of the variation appeared to have provided opportunity for staff working on wards with better results to share their therapeutic practice with staff working on poorer-scoring wards. For example, one site document referred to staff discussion around good therapeutic practice having been tiggered by data showing that service users on the older adult acute inpatient ward had given a higher rating than service users on the general adult acute wards for the quality of therapeutic engagement they received:

*The identification of these differences has led (the team) to explore with ward managers how good practice on collaborative therapeutic working can be shared and trialled across wards.* (Document – nurse director/senior clinician, ID 07).

### 3.2 Opportunity

#### 3.2.1 Organisational barriers confronted

The COVID-19 pandemic was the most frequently discussed organisational barrier in all sites in project facilitation meetings. It was reported that the pandemic had magnified many existing contextual challenges for therapeutic engagement in acute inpatient wards, including higher rates of admission and discharge, increased acuity and complexity of admissions, depleted permanent staff, and increased use of agency staff. The main service adaption mentioned was the creation of specific wards for service users with confirmed or suspected infection. Stakeholders in some sites talked about reduced face-to-face contact because of infected service users being confined to their rooms. Nevertheless, despite this context, introduction of the therapeutic engagement tool seemed to act as a consciousness raiser about organisational constraints faced by registered mental health nurses in delivering therapeutic engagement. In response, several senior stakeholders were prompted to address some of the contextual barriers to therapeutic engagement practice through feasible supportive and remedial actions. In one site, documents referenced efforts involving strategic discussions with registered mental health nurses around the changes needed to provide sufficient time and emotional resources for them to develop good therapeutic relationships. In particular, less focus on routine housekeeping tasks and electronic service user record completion that pulled nurses away from direct service user care:

*The Therapeutic Engagement Questionnaire has empowered registered mental health nurses to re-establish patient engagement as a valued priority…has given staff an opportunity to pause and reflect on nursing as a profession. Registered mental health nurses have let managers know how they feel the profession has become task-oriented with limited time allocated to meaningful service user engagement.* (Document – nurse director/senior clinician, ID 04).

Similarly in project facilitation meetings in another site, stakeholders talked about using Therapeutic Engagement Questionnaire data the site had collected to demonstrate to senior management the negative consequences of registered mental health nurses taking time away from the wards to attend multi-disciplinary team meetings. This was shown to impact on the nurses’ ability to meet service users’ recovery needs, as evidenced in particular by poor service user scores for registered mental health nurses ‘giving them support’ and ‘working in partnership to achieve their goals’. As a consequence, it was decided to reduce the duration of multi-disciplinary team meetings, enabling nurses to spend more time interacting with service users and supporting their needs as they move towards discharge:

*Now more minded of the need to spend more time with patients and less time with MDTs* (multi-disciplinary teams) *and ward reviews.* (Project facilitation meeting – ward manager, ID 14).

The therapeutic engagement tool also appears to have prompted organisational changes in nurse staffing. Documents in one site referred to Therapeutic Engagement Questionnaire data highlighting the value of therapeutic engagement for service user recovery and how this had influenced the implementation of a planned new staffing model that *‘increased numbers of senior and experienced registered mental health nurses on each ward, supporting quality of care delivery… and promoting the importance and value of therapeutic engagement’* (Document – nurse director/senior clinician, ID 04). On one ward of another site, *‘as a direct result (of the therapeutic engagement tool), the most skilled nurses are now allocated to the most unwell patients’* (Document – nurse director/senior clinician, ID 16).

#### 3.2.2 Ward-level practice and culture changes

Having identified through the use of the Therapeutic Engagement Questionnaire that some service users did not know their named (primary) nurse, the ward manager of one study site was persuaded to introduce a new practice model that involved the named nurse introducing themselves to the service user at each twice-daily nurse handover. The nurse also asked the service user open-ended questions about their care. In a project facilitation meeting, it was reported that subsequent ward-level improvements in service user scores for the Therapeutic Engagement Questionnaire statements ‘cares about my feelings, issues, and fears and responds appropriately’ and ‘promotes caring relationships’ had led to plans to introduce the model more widely across wards. Faced with a similar problem, the ward manager of another site described in a project facilitation meeting how they had put cards in the room of each service user with the name of both their consultant and their named nurse. Additionally, consistent use of the term ‘named nurse’ was said to have been encouraged on the ward leading to positive change:

*There is more engagement between service users and their named nurse and even relatives now ask to speak with the named nurse which is something new.* (Project facilitation meeting – ward manager, ID 09).

Several sites reported in project facilitation meetings that their Therapeutic Engagement Questionnaire data showed service users were less involved in planning their care than rated by registered mental health nurses. This had resulted in the organisations initiating nursing practice improvements to help increase service user engagement in care planning. In one site, senior stakeholders reported that the largest disparities were for the statements ‘supports me when I take deliberate and planned care risks and step out of my care comfort zone’, ‘helps me to have control over my care plan’, and ‘works with me to plan my care in advance of me being unwell’. Nursing mangers in the site led reflective sessions with service users and registered mental health nurses on increasing meaningful service user collaboration in care planning. These discussions identified that nurses used inconsistent language and language often not understood by service users when talking about care plans. Consequently, nurses were persuaded to use more service user-centred language and engage in more shared discussion with service users around care planning.

*Ward teams and locality leads offered a series of focus groups open to service users and staff, to collectively consider the issues of communication with a particular interest on how TE (therapeutic engagement) could further inform person-centred care, choice, ownership, and use of language within care plans.* (Document – nurse director/senior clinician, ID 04).

Similarly, in project facilitation meetings in another site, stakeholders referred to locally collected Therapeutic Engagement Questionnaire data that showed a higher proportion of nurses than service users agreed that ‘named nurses’ work with service users in planning their care. In response, a senior stakeholder held a workshop with the ward managers to generate and to discuss change ideas. Scheduled protected engagement times, when named nurses can focus on care planning with service users, was one document-reported improvement change subsequently put into practice:

*Ensuring named nurse sessions are protected times and scheduled within the shift.* (Document – nurse director/senior clinician, ID 16).

### 3.3 Motivation

#### 3.3.1 Awareness of registered mental health nurse influence on service user recovery

Across the study sites, registered mental health nurses’ motivation for therapeutic engagement appears to have been stimulated by local site Therapeutic Engagement Questionnaire data generating awareness of the extent to which their therapeutic work influenced service user recovery. Stakeholders talked in project facilitation meetings about the data making transparent that the higher the score given by service users to the quality of the therapeutic work of their ‘named’ nurse’ or ‘nursing staff’ overall, the higher their rating of the nurse contribution to their recovery, with the converse also seen to apply. Several stakeholders reported that evidence of the positive impact of registered mental health nurses’ engagement work had helped nurses understand the value of their ‘therapeutic power’, providing justification and increasing enthusiasm for therapeutic engagement with service users.

*Feedback from service users has made registered mental health nurses more aware than they had previously thought or recognised of positive interactions with service users. This has served to increase interactions and named nurse sessions.* (Project facilitation meeting – nurse director/senior clinician, ID 01).

In project facilitation meetings in all sites, stakeholders also reported Therapeutic Engagement Questionnaire data that showed registered mental health nurses and service users could have very different perceptions about the extent to which nurses contributed to service user recovery. For example, in one site, registered mental health nurses were reported to underestimate the value of their therapeutic work, as service users gave noticeably higher ratings to the contribution of nurses to their recovery than nurses themselves. This had prompted reflective discussion between ward managers and nurses about the importance of the nurse role and its value in supporting service user recovery:

*It's nurses not valuing what they do. The service users value what nurses do…rekindled discussion and questions about what nursing is about.* (Project facilitation meeting – nurse director/senior clinician, ID 01).

Conversely, in another site, stakeholders talked about registered mental health nurses having initially given markedly higher scores than service users for the contribution of both the named nurse and nursing staff to service user recovery. They also discussed the questionnaire data having highlighted the specific statements where there was most disparity: ‘support in making choices and planned care risks’, ‘working in partnership’, and ‘coordinated and collaborative care planning’. Such explicit evidence was said to have prompted reflective ward-level discussion among nursing staff with managers on how best to address the issues raised.

#### 3.3.2 Alertness to therapeutic work

All sites reported in project facilitation meetings that their Therapeutic Engagement Questionnaire data revealed notable differences in the ratings service users gave for the contribution of their named nurse to their recovery and how they scored statements on the quality of therapeutic engagement delivered. In some sites, stakeholders mentioned that reflective discussions at ward level about these variations had prompted registered mental health nurses to be more aware and alert to their therapeutic engagement work.

*Some variation in how service users see their care which will make registered mental health nurses more conscious of their TE* (therapeutic engagement)*.* (Project facilitation meeting – nurse director/senior clinician ID 17)

#### 3.3.3 Connection with nursing values

Several stakeholders commented on registered mental health nurses being motivated by the therapeutic engagement tool because it aligned with and gave prominence to the core nursing profession values of care and recovery. However, in two case study sites, stakeholders reported in project facilitation meetings that the tool did not accord with the values of some nurses; they were said to be unmotivated by the recognition Therapeutic Engagement Questionnaire data gave their profession for contribution to service user recovery. Senior stakeholders in one site postulated that these feelings were linked to recent disruptive and violent events on a particular ward, leading to increased nurse focus on safety and restraint.

## 4 Discussion

This improvement project sought to identify and understand the acute mental health inpatient organisational and nursing behaviour changes associated with the Therapeutic Engagement Questionnaire, within the context they occurred. To the best of our knowledge, ours is the first theory-based project to understand the behaviour changes associated with a therapeutic engagement tool in multi-site acute mental health inpatient wards. The application of an evidence-based behaviour change model, Capability Opportunity Motivation-Behaviour (Michie et al., 2011a), to an existing complex therapeutic engagement improvement tool has also enabled systematic identification and examination of behaviour change techniques. We suggest several behaviour change techniques and functions linked to the Therapeutic Engagement Questionnaire brought together in a conceptual framework that builds on the Capability Opportunity Motivation-Behaviour model (Table 2).

Findings from our cross-case study behavioural analysis revealed that all three components of the Capability Opportunity Motivation-Behaviour model operated as levers of change: capability, opportunity, and motivation. For therapeutic engagement to occur between registered mental health nurses and service users, nurses must have the capability to engage. Psychological capability (knowledge and skills) was the Capability Opportunity Motivation-Behaviour model component identified as necessary for nurses to improve and thereby have the capacity for therapeutic engagement work with service users. Existing evidence suggests that nurses need guidance to engage therapeutically with service users (McCrae, 2014), whether they possess engagement skills but lack the confidence to practice them or have inadequate skills (McAllister et al., 2019). Nurses are also constrained by the limited theoretical basis for effecting therapeutic interactions (Zugai et al., 2015). An important theme to emerge in our study was use of the Therapeutic Engagement Questionnaire as an evidence base to draw on to build the therapeutic engagement knowledge and skills of registered mental health nurses. The therapeutic engagement statements in the Therapeutic Engagement Questionnaire were an influential factor, employed as a training mechanism or in clinical service supervision by individual ward managers in different case study sites. Although knowledge uptake and application is seldom straightforward, researchers have found that clinical service supervision and education can strengthen nurses’ capacity to develop therapeutic relationships (Roviralta-Vilella et al., 2019).

Sharing of good practice in therapeutic engagement was identified as another sub-theme linked to psychological capability. In some case study sites, senior stakeholders used the reported variation in service user ratings of registered mental health nurses between wards as an opportunity to disseminate examples of therapeutic engagement practice from the highest-scoring wards for nurse training purposes. Such knowledge sharing potentially helped address the widely reported deficiency in support for nurses to develop and maintain therapeutic relationships (Delaney et al., 2022). The top-down attempt to effect behaviour change may also have helped build cultural legitimacy for registered mental health nurse therapeutic engagement work.

Behaviour change also requires opportunity for the behaviour to occur in terms of a conducive physical and social environment (Michie et al., 2011a). The importance of the acute inpatient nursing practice environment in helping forge high-quality therapeutic relationships is highlighted in the literature (Roviralta-Vilella et al., 2019). Good practice requires that nurses prioritise spending therapeutic engagement time with service users over administrative duties (Gabrielsson et al., 2016). Yet acute inpatient nurses experience considerable tensions around keeping often severely mentally-distressed service users safe whilst trying to build and sustain therapeutic relationships and milieu (Simpson, 2022). Nurses may be motivated to seek opportunities for therapeutic encounters with service users to create a basis for therapeutic work (Thibeault, 2016) but, without an organisational focus on the value of the therapeutic relationship, can frequently be diverted to relationships based more on risk management and correction (Bolsinger et al., 2020; Zugai et al., 2015).

The Therapeutic Engagement Questionnaire was introduced during a time of considerable organisational upheaval, with severe clinical pressures associated with the COVID-19 pandemic. Existing contextual pressures were reported to be magnified by the pandemic, in particular, time pressures, task-oriented cultures, and staff shortages. Nevertheless, based on our findings, we suggest that the therapeutic engagement tool had the ability to influence both physical and social opportunities for registered mental health nurses to engage through environmental restructuring and enablement, and social support. Physical opportunities were attempted through top-down efforts to change ward culture by addressing organisational time and resource barriers. These change efforts were led by senior stakeholders, for example, by convening meetings with registered mental health nurses to discuss how nurses could be assisted in establishing therapeutic engagement as a priority on the wards, recommended in the literature as necessary for a good nursing practice environment (Gabrielsson et al., 2016). The resultant changes included less focus on completion of the electronic service user record and reduced time spent on multi-disciplinary team meetings and ward reviews. The therapeutic engagement tool also influenced some senior stakeholders to change the nursing skill mix. On some inpatient wards, for example, more experienced registered mental health nurses were allocated to the most unwell service users. The impact of acute inpatient nurse experience on capacity to engage effectively has been identified in the literature (Roviralta-Vilella et al., 2019).

Efforts to create social opportunities for engagement were both top-down and bottom-up. Existing evidence highlights how staff in acute mental health leadership roles influence change delivery (Laker et al., 2014), yet, in this project, change in leadership was not confined to nurse directors or senior clinicians. Senior stakeholders in some sites were particularly active in facilitating nursing practice improvements to encourage service user engagement in care planning. Their efforts included reflective sessions and workshops with registered mental health nurses, which generated ideas such as using more service user-centred language. Reflective practice has been shown to help change nursing attitudes in the context of therapeutic relationships (Tolosa-Merlos et al., 2023a). Additionally, in a few sites, ward managers also identified and championed changes. These changes included the introduction of new practice models to increase service user awareness and understanding of the therapeutic engagement role of their ‘named nurse’. Evidence suggests a supportive ward culture with a shared vision around therapeutic engagement can positively affect engagement (McAllister et al., 2019).

Nurses’ motivational feelings and attitudes towards therapeutic engagement are known to strongly influence whether or not they engage with service users (McAllister et al., 2019). We found that the Therapeutic Engagement Questionnaire triggered several interrelated automatic and reflective motivational behaviours (Michie et al., 2011a). Knowledge about registered mental health nurses’ impact on service user recovery was a major theme to emerge from this project. Stakeholders reported that Therapeutic Engagement Questionnaire data had helped nurses appreciate their ‘therapeutic power’. This was because the data showed that from the service user perspective, the state of the therapeutic relationship between nurse and service user directly influenced recovery. Additionally, reflective ward-level discussions between managers and nursing staff seem to have been instrumental in building registered mental health nurses’ collective awareness of the value of their therapeutic labour. This is in line with existing evidence that shows registered mental health nurses’ self-confidence and attitude in relation to therapeutic engagement practice is influenced by their awareness of the value to service users of their relational competence (Tolosa-Merlos et al., 2023b).

These motivational components of the therapeutic engagement tool may also have instinctively reminded some registered mental health nurses of their professional nursing values, reinforcing the core registered mental health nursing goal of service user recovery (Coffey et al., 2019) by placing therapeutic engagement more front-of-mind. However, there were other registered mental health nurses for whom automatic motivational factors worked against the desired therapeutic relationship behaviour. There did not seem to be a connection for them between the task of therapeutic engagement and their nursing values; safety and restraint were likely to be more imperative. Since registered mental health nurses perceive the potential for physical violence as high, they can instinctively focus their work on risk assessment rather than therapeutic engagement (McAllister et al., 2019), although a poor therapeutic relationship is associated with increased risk of violence (Bolsinger et al., 2020).

### 4.1 Limitations

The volunteer self-selecting case study sites may have differed in context from sites that did not participate or where use of the therapeutic engagement tool was more problematic. However, inclusion of multiple case study sites across different regions of England is likely to have minimised any partiality that may have arisen. The project was undertaken during the COVID-19 pandemic; therefore, the project researchers were unable to directly observe the behaviour changes and were dependent on reported responses to the therapeutic engagement tool. The project researchers were also unable to observe the cultures and contexts of the wards. One of the project researchers (MC) led development of the tool, which may have influenced the data interpretation. We tried to mitigate this through a team approach to analysis and manuscript drafting. Another limitation is that we collected no evidence to indicate whether or not the behaviour changes would become self-sustaining to effect service user outcomes. A longitudinal study is planned to evaluate the impact of the therapeutic engagement tool on service user outcomes, linking Therapeutic Engagement Questionnaire data with acute inpatient mental healthcare organisations’ routinely collected key performance data.

## 5 Conclusion

Improving therapeutic engagement in order to enhance service user care and safety is integral to the agenda of policymakers and acute mental health hospital organisations in many countries globally. Yet evidence shows that therapeutic engagement in acute mental health inpatient settings remains sub-optimal, impacting negatively on quality of care. This quality improvement project indicated that a therapeutic engagement tool, the Therapeutic Engagement Questionnaire, can prompt positive behaviour changes at the organisation and ward level and to the ways individual registered mental health nurses therapeutically engage with service users, to help strengthen therapeutic engagement practice and potentially improve care quality. Behaviour change was most evident in sites where change leaders were active at nurse director or senior clinician and ward level, enabling physical and social opportunities for therapeutic engagement to take place, reflective discussion and training around practice improvements, and a shared ward vision for therapeutic engagement. The project findings also offer insights and theoretical framing of the behaviour change techniques associated with the therapeutic engagement tool, providing understanding of how the tool can address the different components of therapeutic engagement behaviour: capability (psychological), opportunity (physical and social), and motivation (reflective and automatic).

## Funding sources

Francesca Taylor and Mary Chambers are supported by the National Institute for Health Research (NIHR) Applied Research Collaboration South London (NIHR ARC South London) at King's College Hospital NHS Foundation Trust. The views expressed are those of the authors and not necessarily those of the NIHR or the Department of Health and Social Care.

## Data availability

The datasets used and analysed during the current study are available from the corresponding author on reasonable request.

## CRediT authorship contribution statement

**Francesca Taylor:** Writing – review & editing, Writing – original draft, Methodology, Formal analysis, Data curation, Conceptualization. **Sarah Galloway:** Writing – review & editing, Project administration. **Kris Irons:** Writing – review & editing, Project administration. **Lorna Mess:** Writing – review & editing, Project administration. **Laura Pemberton:** Writing – review & editing, Project administration. **Karen Worton:** Writing – review & editing, Project administration. **Mary Chambers:** Writing – review & editing, Methodology, Formal analysis, Conceptualization.

## Declaration of competing interest

The authors declare that they have no known competing financial interests or personal relationships that could have appeared to influence the work reported in this paper.

## References

[bib0001] Bolsinger J., Jaeger M., Hoff P., Theodoridou A. (2020). Challenges and opportunities in building and maintaining a good therapeutic relationship in acute psychiatric settings: a narrative review. Front. Psychiatry.

[bib0002] Boyatzis R.E. (1998). Transforming Qualitative Information: Thematic Analysis and Code Development.

[bib0003] Brett J., Staniszewska S., Mockford C. (2014). Mapping the impact of patient and public involvement on health and social care research: a systematic review. Health Expect..

[bib0004] Chambers M. (1992). Psychiatric and Mental Health Nursing: Theory and Practice.

[bib0005] Chambers M., McAndrew S., Nolan F., Thomas B., Watts P., Kantaris X. (2017). Service user involvement in the coproduction of a mental health nursing metric: the therapeutic engagement questionnaire. Health Expect..

[bib0006] Chambers M., McAndrew S., Nolan F. (2019). The therapeutic engagement questionnaire (TEQ): a service-user focused mental health nursing outcome measure. BMC Psychiatry.

[bib0007] Chambers M., McAndrew S., Nolan F., Thomas B., Watts P., Kantaris X. (2021). Measuring therapeutic engagement in acute mental health inpatient environments: the perspectives of service users and mental health nurses. BMC Psychiatry.

[bib0008] Coffey M., Hannigan B., Barlow S. (2019). Recovery-focused mental health care planning and co-ordination in acute inpatient mental health settings: a cross national comparative mixed methods study. BMC Psychiatry.

[bib0009] Cutliffe J.R., Santos J.C., Kozel B., Taylor P., Lees D. (2015). Raiders of the lost art: a review of published evaluations of inpatient mental health care experiences emanating from the United Kingdom, Portugal, Canada, Switzerland, Germany, and Australia. Int. J. Ment. Health Nurs..

[bib0010] Davis R., Campbell R., Hildon Z., Hobbs L., Michie S. (2015). Theories of behaviour and behaviour change across the social and behavioural sciences: a scoping review. Health Psychol. Rev..

[bib0011] Delaney K.R., Loucks J., Ray R. (2022). Delineating quality indicators of inpatient psychiatric hospitalization. J. Am. Psychiatr. Nurse Assoc..

[bib0012] Department of Health and Social Care. (2023). https://www.gov.uk/government/calls-for-evidence/mental-health-and-wellbeing-plan-discussion-paper-and-call-for-evidence.

[bib0013] Dodd E., Cheston R., Proctor C. (2018). Protected engagement time on older adult mental health wards: a thematic analysis of the views of patients, carers, and staff. Int. J. Ment. Health Nurs..

[bib0014] Ethnicity Facts and Figures Service (2022). Regional Ethnic Diversity. Ethnic Groups by Region and Ethnic Diversity by Local Authority.

[bib0015] Flückiger C., Del Re A.C., Wlodasch D., Horvath A.O., Solomonov N., Wampold B.E. (2020). Assessing the alliance-outcome association adjusted for patient characteristics and treatment processes: a meta-analytic summary of direct comparisons. J. Couns. Psychol..

[bib0016] Gabrielsson S., Sävenstadt S., Olsson M. (2016). Taking personal responsibility: nurses’ and assistant nurses’ experiences of good nursing practice in psychiatric inpatient care. Int. J. Ment. Health Nurs..

[bib0017] Hartley S., Raphael J., Lovell K., Berry K. (2020). Effective nurse-patient relationships in mental health care: a systematic review of interventions to improve the therapeutic alliance. Int. J. Nurs. Stud..

[bib0018] Holloway I., Galvin K. (2016). Qualitative Research in Nursing and Health Care.

[bib0019] Johnson S., Dalton-Locke C., Baker J. (2022). Acute psychiatric care: approaches to increasing the range of services and improving access and quality of care. World Psychiatry.

[bib0020] Kelly L.M., Cordeiro M. (2020). Three principles of pragmatism for research on organizational processes. Method. Innov..

[bib0021] Kingston University London. (2023). https://www.kingston.ac.uk/faculties/faculty-of-health-social-care-education/research/cahscr/mental-health/therapeutic-engagement-questionnaire.

[bib0022] Kingston University London (2023). https://d68b3152cf5d08c2f050-97c828cc9502c69ac5af7576c62d48d6.ssl.cf3.rackcdn.com/documents/user-upload/kingston-university-23cfa370447-kingston-university-69314b138b2.pdf.

[bib0023] Laker C., Callard F., Flach C., Williams P., Sayer J., Wykes T. (2014). The challenge of change in mental health services: measuring staff perceptions of barriers to change and their relationship to job status and satisfaction using a new measure (VOCALISE). Implement. Sci..

[bib0024] Le Boutillier C., Chevalier A., Lawrence V. (2015). Staff understanding of recovery-orientated mental health practice: a systematic review and narrative synthesis. Implement. Sci..

[bib0025] Marshall M., de Silva D., Cruickshank L., Shand J., Wei L., Anderson J. (2017). What we know about designing an effective improvement intervention (but too often fail to put into practice). BMJ Qual. Saf..

[bib0026] Martin G.P., Williams O. (2019). What Works Now? Evidence Informed Policy and Practice.

[bib0027] McAllister S., Robert G., Tsianakas V., McCrae N. (2019). Conceptualising nurse-patient therapeutic engagement on acute mental health wards: an integrative review. Int. J. Nurs. Stud..

[bib0028] McAllister S., Simpson A., Tsianakas V., Robert G. (2021). ‘What matters to me’: a multi-method qualitative study exploring service users’, carers’ and clinicians’ needs and experiences of therapeutic engagement on acute mental health wards. Int. J. Ment. Health Nurs..

[bib0029] McAllister S., Simpson A., Tsianakas V. (2021). Developing a theory-informed complex intervention to improve nurse-patient therapeutic engagement employing experience-based co-design and the behaviour change wheel: an acute mental health ward case study. British Medical Journal Open.

[bib0030] McAndrew S., Chambers M., Nolan F., Thomas B., Watts P. (2014). Measuring the evidence: reviewing the literature of the measurement of therapeutic engagement in acute mental health inpatient wards. Int. J. Ment. Health Nurs..

[bib0031] McCrae N. (2014). Protected engagement time in mental health inpatient units. Nurs. Manag..

[bib0032] Michie S., van Straten M.M., West R. (2011). The behaviour change wheel: a new method for characterising and designing behaviour change interventions. Implement. Sci..

[bib0033] Michie S., Abrahams C., Eccles M.P., Francis J.J., Hardeman W., Johnson M. (2011). Strengthening evaluation and implementation by specifying components of behaviour change interventions: a study protocol. Implement. Sci..

[bib0034] Michie S., Richardson M., Johnson M. (2013). The behaviour change technique taxonomy (v1) of 93 hierarchically clustered techniques: building an international consensus for the reporting of behaviour change interventions. Ann. Behav. Med..

[bib0035] Molin J., Lindgren B.M., Graneheim U.H., Ringer A. (2018). Time together: a nursing intervention in psychiatric inpatient care: feasibility and effects. Int. J. Ment. Health Nurs..

[bib0036] Moreno-Poyato A.R., Casanov-Gavrigos G., Roldàn-Merino J.F., Rodríguez-Nogueira O., MiRTCIME.CAT working group. (2021). Examining the association between evidence-based practice and the nurse-patient therapeutic relationship in mental health units: a cross-sectional study. J. Adv. Nurs..

[bib0037] National Mental Health Commission (2020). https://www.mentalhealthcommission.gov.au/getmedia/28fe94a6-ae18-47d3-bd62-ad8a048b8582/NMHC_Vision2030_ConserviceuserltationReport_March2020.pdf.

[bib0038] O'Brien B.C., Harris I.B., Beckman T.J. (2014). Standards for reporting qualitative research: a synthesis of recommendations. Acad. Med..

[bib0039] Office for National Statistics (2015). https://geoportal.statistics.gov.uk/datasets/ons::rural-urban-classification-2011-of-nuts-3-2015-in-england-1/about.

[bib0040] Organisation for Economic Co-operation and Development Health Policy Studies (2021). https://www.oecd.org/health/a-new-benchmark-for-mental-health-systems-4ed890f6-en.htm.

[bib0041] Patel V., Saxena S., Lundt C. (2018). The Lancet Commission on global mental health and sustainable development. Lancet.

[bib0042] Pope C., Mays N. (2020).

[bib0043] Raphael J., Price O., Hartley S., Haddock G., Bucci S., Berry K. (2021). Overcoming barriers to implementing ward-based psychosocial interventions in acute inpatient mental health settings: a meta-synthesis. Int. J. Nurs. Stud..

[bib0044] Roviralta-Vilella M., Moreno-Poyato A.R., Rodríguez-Nogueira O., Duran-Jordà X., Roldàn-Merino J.F., on behalf of MiRTCIME.CAT working group (2019). Relationship between the nursing practice environment and the therapeutic relationship in acute mental health units: a cross-sectional study. Int. J. Ment. Health Nurs..

[bib0045] Simpson A. (2022). Activities and technologies: developing safer acute inpatient mental health care. World Psychiatry.

[bib0046] Staniszewska S., Mockford C., Chadburn G. (2019). Experiences of in-patient mental health services: systematic review. Br. J. Psychiatry.

[bib0047] Taylor F., Galloway S., Irons K. (2022). Barriers and enablers to implementation of the therapeutic engagement questionnaire in acute mental health inpatient wards in England: a qualitative study. Int. J. Ment. Health Nurs..

[bib0048] Thibeault C. (2016). An interpretation of nurse-patient relationships in inpatient psychiatry: understanding the mindful approach. Glob. Qual. Nurs. Res..

[bib0049] Tolosa-Merlos D., Moreno-Poyato A.R., González-Palau F. (2023). The therapeutic relationship at the heart of nursing care: a participatory action research in acute mental health units. J. Clin. Nurs..

[bib0050] Tolosa-Merlos D., Moreno-Poyato A.R., González-Palau F. (2023). Exploring the therapeutic relationship through reflective practice of nurses in acute mental health units: a qualitative study. J. Clin. Nurs..

[bib0051] Wyder M., Bland R., Blyth A., Matarasso B., Crompton D. (2015). Therapeutic relationships and involuntary treatment order: service users’ interactions with health-care professionals on the ward. Int. J. Ment. Health Nurs..

[bib0052] Wykes T., Csipke E., Williams P. (2018). Improving patient experiences of mental health inpatient care: a randomised controlled trial. Psychol. Med..

[bib0053] Yin R.K. (2018). Case Study Research and Applications: Design and Methods.

[bib0054] Zugai J.S., Stein-Parbury J., Roche M. (2015). Therapeutic alliance in mental health nursing: an evolutionary concept analysis. Issues Ment. Health Nurs..

